# Chemoradiotherapy for locally advanced head and neck cancer: 10-year follow-up of the UK Head and Neck (UKHAN1) trial

**DOI:** 10.1016/S1470-2045(09)70306-7

**Published:** 2009-09-09

**Authors:** Jeffrey S Tobias, Kathryn Monson, Nirmal Gupta, Hugh MacDougall, John Glaholm, Iain Hutchison, Latha Kadalayil, Allan Hackshaw, on behalf of the UK Head and Neck Cancer Trialists' Group

**Affiliations:** a Department of Clinical Oncology, University College Hospital, London, UK; b Cancer Research UK Sussex Psychosocial Oncology Group, Brighton and Sussex Medical School, Brighton, UK; c Department of Clinical Oncology, The Christie Hospital, Manchester, UK; d Faculty of Medicine, Bute Medical School, University of St Andrews, St Andrews, UK; e Department of Oncology, Queen Elizabeth Hospital Birmingham, Birmingham, UK; f Department of Oral and Maxillofacial Surgery, St Bartholomew's Hospital, London, UK; g Cancer Research UK and UCL Cancer Trials Centre, University College London, London, UK

## Abstract

**Background:**

Between 1990 and 2000, we examined the effect of timing of non-platinum chemotherapy when combined with radiotherapy. We aimed to determine whether giving chemotherapy concurrently with radiotherapy or as maintenance therapy, or both, affected clinical outcome. Here we report survival and recurrence after 10 years of follow-up.

**Methods:**

Between Jan 15, 1990, and June 20, 2000, 966 patients were recruited from 34 centres in the UK and two centres from Malta and Turkey. Patients with locally advanced head and neck cancer, and who had not previously undergone surgery, were randomly assigned to one of four groups in a 3:2:2:2 ratio, stratified by centre and chemotherapy regimen: radical radiotherapy alone (n=233); radiotherapy with two courses of chemotherapy given simultaneously on days 1 and 14 of radiotherapy (SIM alone; n=166); or 14 and 28 days after completing radiotherapy (SUB alone, n=160); or both (SIM+SUB; n=154). Chemotherapy was either methotrexate alone, or vincristine, bleomycin, methotrexate, and fluorouracil. Patients who had previously undergone radical surgery to remove their tumour were only randomised to radiotherapy alone (n=135) or SIM alone (n=118), in a 3:2 ratio. The primary endpoints were overall survival (from randomisation), and event-free survival (EFS; recurrence, new tumour, or death; whichever occurred first) among patients who were disease-free 6 months after randomisation. Analyses were by intention to treat. This trial is registered at www.Clinicaltrials.gov, number NCT00002476.

**Findings:**

All 966 patients were included in the analyses. Among patients who did not undergo surgery, the median overall survival was 2·6 years (99% CI 1·9–4·2) in the radiotherapy alone group, 4·7 (2·6–7·8) years in the SIM alone group, 2·3 (1·6–3·5) years in the SUB alone group, and 2·7 (1·6–4·7) years in the SIM+SUB group (p=0·10). The corresponding median EFS were 1·0 (0·7–1·4), 2·2 (1·1–6·0), 1·0 (0·6–1·5), and 1·0 (0·6–2·0) years (p=0·005), respectively. For every 100 patients given SIM alone, there are 11 fewer EFS events (99% CI 1–21), compared with 100 given radiotherapy, 10 years after treatment. Among the patients who had previously undergone surgery, median overall survival was 5·0 (99% CI 1·8–8·0) and 4·6 (2·2–7·6) years in the radiotherapy alone and SIM alone groups (p=0·70), respectively, with corresponding median EFS of 3·7 (99% CI 1·1–5·9) and 3·0 (1·2–5·6) years (p=0·85), respectively. The percentage of patients who had a significant toxicity during treatment were: 11% (radiotherapy alone, n=25), 28% (SIM alone, n=47), 12% (SUB alone, n=19), and 36% (SIM+SUB, n=55) among patients without previous surgery; and 9% (radiotherapy alone, n=12) and 20% (SIM alone, n=24) among those who had undergone previous surgery. The most common toxicity during treatment was mucositis. The percentage of patients who had a significant toxicity at least 6 months after randomisation were: 6% (radiotherapy alone, n=13), 6% (SIM alone, n=10), 4% (SUB alone, n=7), and 6% (SIM+SUB, n=9) among patients who had no previous surgery; and 7% (radiotherapy alone, n=10) and 11% (SIM alone, n=13) among those who had undergone previous surgery. The most common toxicity 6 months after treatment was xerostomia, but this occurred in 3% or less of patients in each group.

**Interpretation:**

Concurrent non-platinum chemoradiotherapy reduces recurrences, new tumours, and deaths in patients who have not undergone previous surgery, even 10 years after starting treatment. Chemotherapy given after radiotherapy (with or without concurrent chemotherapy) is ineffective. Patients who have undergone previous surgery for head and neck cancer do not benefit from non-platinum chemotherapy.

**Funding:**

Cancer Research UK, with support from University College London and University College London Hospital Comprehensive Biomedical Research Centre.

## Introduction

Head and neck cancers are relatively common (about 7500 new cases in the UK and 45 000 in the US each year), and are increasing in incidence in some countries such as the UK, mainly because of smoking and excessive alcohol intake.^1^, ^2^, ^3^ Standard treatment until 1990 was surgical resection or radical radiotherapy, sometimes both. The role of chemotherapy has been investigated for over 20 years, but with varying reports of effectiveness. Toxicity is especially important since patients are typically unfit, often with coexistent illness.^4^, ^5^

Over the past decade the role of chemotherapy has become clearer. A meta-analysis published in 2000^6^ and updated in 2009^7^ indicated that concomitant chemotherapy reduced the risk of dying by 19% compared with radiotherapy alone. The number of agents found to be active in squamous-cell carcinoma has increased, and the best ways of combining them continue to be investigated.

The UK Head and Neck (UKHAN) cancer group was established in 1990 to investigate the effectiveness of chemotherapy used in conjunction with radiotherapy and surgery. At the time, it was believed that chemotherapy given at the same time as radical radiotherapy (ie, concurrent) and afterwards (ie, maintenance) could be effective. The UKHAN1 trial was designed to determine whether adding chemotherapy to standard radical treatment—radiotherapy with or without primary surgery—would improve survival and loco-regional control. Other objectives were to assess the effect of timing and duration of chemotherapy—ie, whether two courses should be given simultaneously with (SIM alone) or subsequent to (SUB alone) the radiotherapy course, or both simultaneously and following radiotherapy (four courses, SIM+SUB). Earlier results were presented and reported in 2001.^8^ Here we report the results after 10 years of follow-up.

## Methods

### Patients

Patients with locally advanced squamous-cell carcinoma of the head and neck were included in a factorial randomised trial if they were judged to be suitable for radical radiotherapy as either initial treatment or following an operation (generally patients at high risk of recurrence following surgery due to margin status or advanced stage of disease at presentation).

Before participating in the trial, patients underwent a full clinical head and neck examination, with biopsy or surgical resection of the primary tumour and chest radiography. Patients were eligible if they satisfied the following criteria: age 18 years or over; considered fit enough to receive any of the trial treatments; had histological confirmation of squamous-cell carcinoma, with T2 to T4 primary lesions (including node-negative cases) or were node positive; had full normal blood count, and creatinine and urea levels within normal ranges; showed no evidence of distant metastases; and had no previous treatment for the cancer other than surgical excision. Ethical approval was obtained from each centre. All patients gave written informed consent.

### Procedures

The extent of previous surgery, radiotherapy regimen, and nutritional support with either nasogastric or percutaneous-endoscopic radiologically inserted gastrostomy feeding, were based on local policies to maximise recruitment. Investigators declared their radiotherapy treatment policy prospectively before participating. Surgery before randomisation was also determined by local practice, and could involve resection of the primary tumour alone with or without comprehensive neck-node resection, provided that the intention was to completely clear the tumour. Usually at the outset these surgery patients were scheduled for adjunctive postoperative radiotherapy because of their advanced disease.

Although radical radiotherapy could be given according to local practice, it had to be approved by the trial steering committee, and investigators were asked to adhere to it for all patients within that centre, regardless of treatment allocation. Two extensively tested regimens were recommended^9^, ^10^, ^11^ (webappendix). The Manchester regimen involved a radical course to the primary tumour and lymph nodes in 15 or 16 fractions (five fractions per week) over 3–3·5 weeks. Treatment volume was kept to a minimum, and no attempt was made to encompass the entire regional lymphatic drainage areas. The minimum tumour dose was in the range of 50–55 Gy for a field area of 25–40 cm^2^, reduced to 45 Gy for larger fields. The South-East Co-operative Oncology Group regimen involved irradiation with planned fields to adequately cover the primary tumour in unresected cases, and in most cases the lymph-node drainage area. The recommended fractionation was 1·8–2 Gy daily, 5 days per week, to a minimum total dose of 60 Gy, although higher doses were permitted. Another common regimen, used in Birmingham, Edinburgh, and some other centres, had the following schedule: 55 Gy given in 20 fractions (2·75 Gy per fraction) over 4 weeks to the primary tumour and first station lymphatic drainage, and 41·25 Gy in 15 fractions to the elective neck. 50 Gy given in 20 fractions (2·5 Gy per fraction) was given postoperatively.

Chemotherapy regimens were either methotrexate alone or vincristine, bleomycin, methotrexate, and fluorouracil (VBMF); webappendix.^8^, ^9^ SIM started on days 1 and 14 concurrently with radiotherapy, and SUB started 14 and 28 days after completing radiotherapy. Methotrexate was given intravenously in two doses of 100 mg/m^2^: the first dose was given 24 h before radiotherapy and the second dose was given on day 14 of radiotherapy. Folinic acid rescue was given if serum methotrexate concentration at 24 h after treatment exceeded 0·4 μmol/L.^10^ VBMF consisted of vincristine 1·4 mg/m^2^ (maximum 2 mg), bleomycin 30 mg, fluorouracil 500 mg, and methotrexate 100 mg/m^2^. Drugs were given intravenously by slow bolus injection except bleomycin, which was given by intramuscular injection. Hydrocortisone (100 mg intramuscular injection) could be given to minimise the risk of bleomycin reactions, and anti-emetics were given according to local practice.

Primary endpoints were overall survival, measured from the time from randomisation to death from any cause, and event-free survival (EFS), defined as recurrence, new tumour, or death (whichever occurred first) among patients who were disease-free 6 months after randomisation. Nine patients who had salvage treatment after being disease free, but without a recorded date of recurrence, were counted as a suspected recurrence. Other endpoints were control of local and regional disease (ie, the complete remission rate) at 6 months; time to recurrence; death from head and neck cancer; and toxicity during and after treatment, which was classified as significant if hospitalisation was required during chemoradiation, therapy was required to alleviate chronic treatment-related toxicity (eg, dilatations for oesophageal strictures), or clinicians recorded an event as severe after treatment. Although toxicity was not recorded on a standardised scale during the trial, we compared the reported adverse-event grading and descriptions with the Common Terminology Criteria for Adverse Events (CTCAE) version 3, as part of the statistical analysis. A significant event was generally comparable to a grade 3 or 4 CTCAE event (mostly grade 3). Compliance with radiotherapy or chemotherapy was determined: a non-complier was defined as a patient who did not receive the full dose according to the trial protocol. In October, 2008, an active data chase was completed to ascertain which patients had died or had a recurrence or new tumour, and the date patients were last seen alive.

### Randomisation and masking

Block stratified randomisation was used, and allocations were concealed from investigators and patients by generating random number lists centrally at the coordinating centre (Cancer Research UK and University College London Cancer Trials Centre). Patients were to be allocated to radiotherapy alone, SIM alone, SUB alone or SIM+SUB if they had not had previous surgery, using a block size of nine (allocation ratio 3:2:2:2, which is 1:2 in favour of chemotherapy, to increase the total number of patients given chemotherapy); or only to radiotherapy alone or SIM alone if they had previously undergone surgery, using a block size of five (allocation ratio 3:2). Randomisation stratification factors were centre (which automatically stratifies for radiotherapy regimen and all other local policies for patient management) and chemotherapy regimen (though all but two centres used a single regimen during the trial). Hospital clinicians recruited patients, and centres telephoned the co-ordinating centre, who assigned each patient a treatment allocation after recording the eligibility and stratification factors.

### Statistical analysis

The sample size of about 1000 patients was based on detecting an increase in 5-year survival from 25% in the radiotherapy-alone group to 35% in the chemotherapy groups combined, with 90% power and two-sided 5% level of statistical significance. Statistical analyses were by intention to treat and based on the log-rank test. Hazard ratios (HR) are reported separately for patients who had or had not undergone previous surgery. All p values are two-sided, and 99% CI were used to allow for having multiple comparisons (95% for the primary objective of any chemotherapy versus radiotherapy alone). Patients not disease-free by 6 months after randomisation were counted as having an event at time zero for the EFS analysis. All analyses were done using STATA version 10. This trial is registered with www.Clinicaltrials.gov, number NCT00002476.

### Role of the funding source

The funding organisation (Cancer Research UK) approved the study design but had no involvement in the collection, analysis, or interpretation of the data, or the writing of this report. The corresponding author had full access to all of the data and the final responsibility to submit the manuscript for publication.

## Results

**Figure 1:**
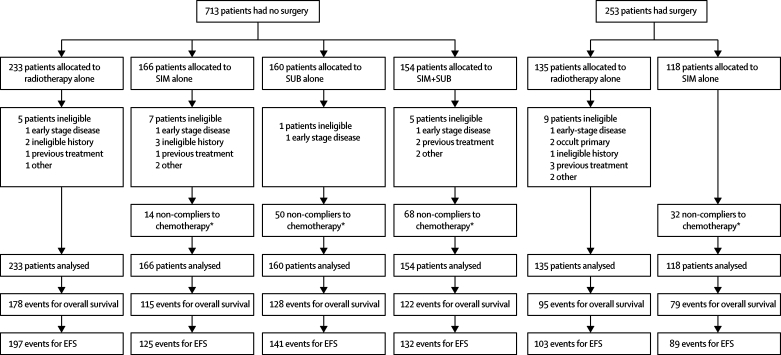
Trial profile EFS=event-free survival. SIM=radiotherapy with two courses of chemotherapy given simultaneously on days 1 and 14 of radiotherapy. SUB= radiotherapy with two courses of chemotherapy given 14 and 28 days after completing radiotherapy. *See table 2. Only 26 patients did not fully meet the eligibility criteria, but they were included in the analysis.

Between Jan 15, 1990, and June 20, 2000, 966 patients were recruited from 34 centres in the UK and two from Malta and Turkey. The median length of follow-up was 10 years after censoring those who had died (maximum of 17 years), with 4397 person-years in total. No data were available for only 89 patients after 2005. The trial profile is shown in figure 1; baseline characteristics are shown in table 1.

**Table 1:** Distribution of baseline characteristics according to trial group

		**No previous surgery (N=713)**				**Surgery (N=253)**	
		Radiotherapy alone (N=233)	SIM alone (N=166)	SUB alone (N=160)	SIM+SUB (N=154)	Radiotherapy alone (N=135)	SIM alone (N=118)
Age (years; median and range)		60 (17–78)	59 (29–78)	60 (34–82)	60(26–84)	56 (32–76)	60 (36–81)
Sex							
	Male	188 (81)	125 (75)	119 (74)	134 (87)	104 (77)	88 (75)
	Female	45 (19)	40 (24)	40 (25)	19 (12)	31 (23)	30 (25)
Tumour stage							
	T1	19 (8)	10 (6)	11 (7)	8 (5)	7 (5)	8 (7)
	T2	106 (46)	72 (43)	64 (40)	56 (36)	22 (16)	29 (25)
	T3	52 (22)	45 (27)	35 (22)	49 (32)	27 (20)	26 (22)
	T4	56 (24)	38 (23)	50 (31)	39 (25)	76 (56)	54 (46)
	Unknown	0	1 (<1)	0	2 (1)	3 (2)	1 (<1)
Nodal status							
	Negative	129 (55)	88 (53)	95 (59)	80 (52)	58 (43)	39 (33)
	Positive	104 (45)	77 (46)	65 (41)	73 (47)	76 (56)	78 (66)
	Unknown	0	1 (<1)	0	1	1 (<1)	1 (<1)
Stage							
	Stage II	64 (27)	47 (28)	47 (29)	36 (23)	6 (4)	10 (8)
	Stage III	71 (30)	53 (32)	40 (25)	49 (32)	26 (19)	25 (21)
	Stage IV	97 (42)	63 (38)	72 (45)	66 (43)	98 (73)	81 (69)
	Unknown	1 (<1)	3 (2)	2 (1)	1 (<1)	5 (4)	2 (2)
Site of primary							
	Larynx	73 (31)	51 (31)	54 (34)	46 (30)	28 (21)	34 (29)
	Oral cavity	41 (18)	18 (11)	20 (13)	19 (12)	46 (34)	43 (36)
	Oropharynx	78 (34)	65 (39)	53 (33)	60 (39)	34 (25)	25 (21)
	Nasopharynx	14 (6)	13 (8)	12 (8)	11 (7)	2 (1)	2 (2)
	Hypopharynx	22 (9)	14 (8)	14 (9)	17 (11)	18 (13)	11 (9)
	Other	5 (2)	5 (3)	7 (4)	1 (<1)	7 (5)	3 (3)
	Unknown	0	0	0	0	1 (<1)	0
Surgery group							
	Margins cleared*	..	..	..	..	64 (47)	56 (47)
	Neck dissection done	..	..	..	..	94 (70)	88 (74)

SIM=radiotherapy with two courses of chemotherapy given simultaneously on days 1 and 14 of radiotherapy. SUB= radiotherapy with two courses of chemotherapy given 14 and 28 days after completing radiotherapy. Data are n (%), unless stated otherwise.

* Primary site resection margins were clear.

7% of patients (69/966) were known to have not completed the planned radiotherapy course (table 2). 27% of patients (164/598) allocated to receive chemotherapy did not complete their full course of treatment, mainly due to disease progression, acute toxicity, or withdrawal from treatment. Among patients without prior surgery, only 8% (14/166) of the SIM alone group were non-compliers to chemotherapy, compared with 31% (50/160) and 44% (68/154) in the SUB alone and SIM+SUB groups respectively. 27% (32/118) of SIM alone patients who had surgery did not complete their chemotherapy.

**Table 2:** Reasons for not complying with the scheduled radiotherapy and chemotherapy regimen specified in the trial protocol (did not receive the full dose, or did not receive the therapy at all)

	**No previous surgery (N=713)**				**Surgery (N=253)**	
	Radiotherapy alone (N=233)	SIM alone (N=166)	SUB alone (N=160)	SIM+SUB (N=154)	Radiotherapy alone (N=135)	SIM alone (N=118)
**Radiotherapy non-compliance**						
Disease progression	3 (1)	4 (2)	1 (<1)	1 (<1)	1 (<1)	3 (3)
Deaths	2 (<1)	3 (2)	0	6 (4)	2 (2)	2 (2)
Treatment-related deaths	1 (<1)	0	0	3 (2)	0	0
Toxicity	2 (<1)	0	0	1 (<1)	0	3 (3)
Patient withdrew	1 (<1)	0	2 (1)	0	2 (2)	0
Other	7 (3)	7 (4)	3 (2)	5 (3)	2 (2)	2 (2)
Data missing	0	0	2 (1)	0	2 (2)	1 (<1)
Total non-compliers*	16 (7)	14 (8)	6 (4)	16 (10)	7 (5)	10 (8)
**Chemotherapy non-compliance**						
Disease progression	NA	4 (2)	11 (7)	7 (5)	NA	4 (3)
Patient not fit enough	NA	0	2 (1)	0	NA	1 (<1)
Deaths	NA	0	6 (4)	8 (5)	NA	1 (<1)
Treatment-related deaths	NA	0	2 (1)	8 (5)	NA	0
Toxicity	NA	3 (2)	8 (5)	24 (16)	NA	14 (12)
Reduced renal function	NA	1 (<1)	4 (3)	7 (5)	NA	0
Patient withdrew	NA	2 (1)	8 (5)	10 (6)	NA	6 (5)
Other	NA	4 (2)	9 (6)	4 (3)	NA	6 (5)
Data missing	NA	0	2 (1)	1 (<1)	NA	2 (2)
Total non-compliers*	NA	14 (8)	50 (31)	68 (44)	NA	32 (27)

SIM=radiotherapy with two courses of chemotherapy given simultaneously on days 1 and 14 of radiotherapy. SUB= radiotherapy with two courses of chemotherapy given 14 and 28 days after completing radiotherapy. NA=not applicable. Data are n (%).

* Excluding those with missing data.

69% (662/966) of patients were disease-free at 6 months (webappendix). Among those without previous surgery, the proportion disease-free at 6 months was highest in the SIM alone group (73%, 122/166) compared with the other groups: radiotherapy alone (63%, 146/233), SUB alone (66%, 106/160), and SIM+SUB (62%, 95/154). There was little difference among patients who had undergone previous surgery: radiotherapy alone (76%, 102/135) and SIM alone (77%, 91/118).

There were 717 deaths among all 966 patients, and 483 EFS events among the 662 patients who were disease free at 6 months (table 3). Estimated 5-year overall survival was 43% (95% CI 38–48) and EFS was 32% (27–37) in the radiotherapy-alone group (patients with or without previous surgery). There was no effect on overall survival or EFS for any chemotherapy (ie, combining SIM alone, SUB alone, and SIM+SUB). Compared with radiotherapy alone, the overall survival and EFS HR for any chemotherapy were 0·97 (95% CI 0·83–1·13, p=0·71) and 0·92 (0·79–1·06; p=0·25), respectively. However, because the effects of SIM and SUB clearly differed, they need to be considered separately. The chemotherapy or radiotherapy regimen used did not affect the results (webappendix).

**Table 3:** The number and causes of death, and the number of first events in patients who were disease free at 6 months after randomisation (used to examine event-free survival), according to trial group

		**No previous surgery (N=713)**				**Surgery (N=253)**	
		Radiotherapy alone (N=233)	SIM alone (N=166)	SUB alone (N=160)	SIM+SUB (N=154)	Radiotherapy alone (N=135)	SIM alone (N=118)
Cause of death							
	Treatment-related*	1 (0·4)	1 (0·6)	2 (1·2)	8 (5·2)	0	0
	Post-operative deaths	2 (0·8)	0	3 (1·9)	0	3 (2·2)	0
	Head and neck cancer	120 (51)	83 (50)	95 (59)	72 (47)	66 (49)	55 (47)
	Not cancer related	47 (20)	25 (15)	25 (16)	34 (22)	22 (16)	21 (18)
	Uncertain	8 (3·4)	6 (3·6)	3 (1·9)	8 (5·2)	4 (3·0)	3 (2·5)
	Total deaths	178	115	128	122	95	79
Disease-free at 6 months†		146 (63)	122 (73)	106 (66)	95 (62)	102 (76)	91 (77)
First event‡							
	Recurrence	57 (39)	39 (32)	50 (47)	35 (37)	23 (22)	25 (27)
	Death	31 (21)	28 (23)	18 (17)	23 (24)	25 (24)	18 (20)
	New tumour	22 (15)	14 (11)	19 (18)	15 (14)	22 (22)	19 (21)
	Total first events	110	81	87	73	70	62

SIM=radiotherapy with two courses of chemotherapy given simultaneously on days 1 and 14 of radiotherapy. SUB= radiotherapy with two courses of chemotherapy given 14 and 28 days after completing radiotherapy. Data are n (%).

* Radiotherapy or chemotherapy.

† Expressed as a percentage of all patients randomised to the group.

‡ Expressed as a percentage of the number disease free at 6 months.

**Figure 2:**
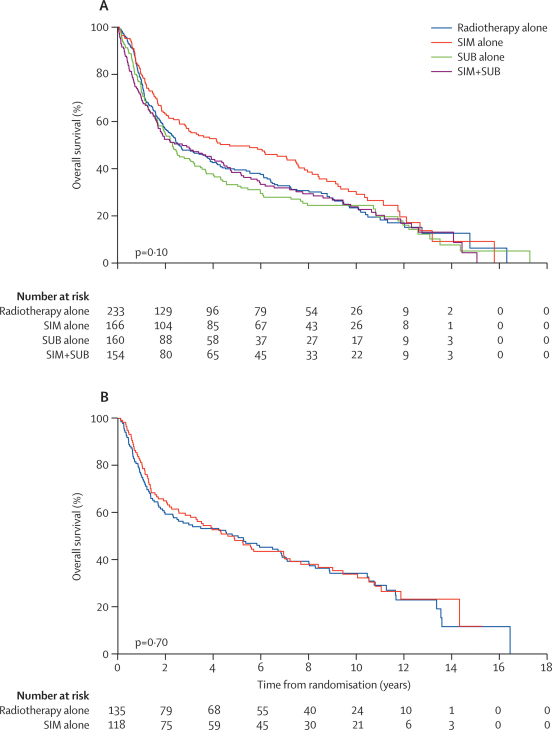
Overall survival according to treatment groups for patients who had no surgery (A) and those who had undergone surgery (B) before randomisation SIM=radiotherapy with two courses of chemotherapy given simultaneously on days 1 and 14 of radiotherapy. SUB= radiotherapy with two courses of chemotherapy given 14 and 28 days after completing radiotherapy.

**Figure 3:**
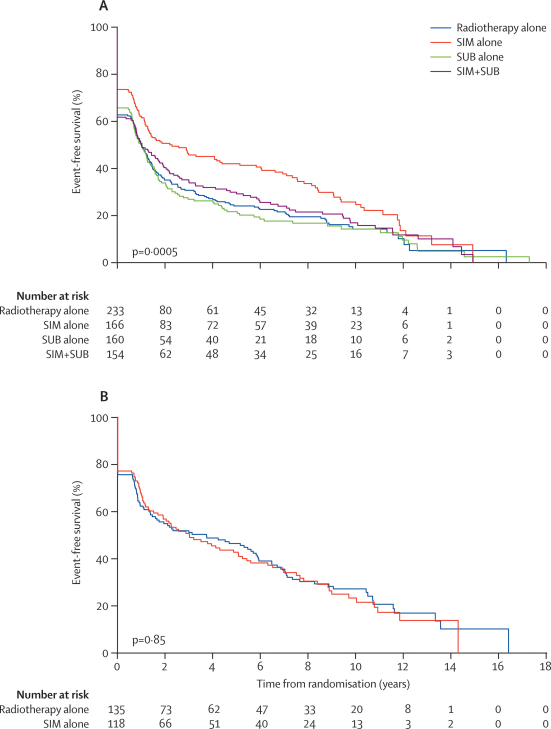
Event-free survival (EFS) according to treatment groups for patients who had no surgery (A) and those who had undergone surgery (B) before randomisation SIM=radiotherapy with two courses of chemotherapy given simultaneously on days 1 and 14 of radiotherapy. SUB= radiotherapy with two courses of chemotherapy given 14 and 28 days after completing radiotherapy.

Kaplan–Meier curves for overall survival and EFS are shown in Figure 2, Figure 3. In patients without previous surgery, median overall survival was 2·6 (99% CI 1·9–4·2) years for those allocated to radiotherapy alone, 4·7 (2·6–7·8) years for SIM alone, 2·3 (1·6–3·5) years for SUB alone, and 2·7 (1·6–4·7) years in those allocated to SIM+SUB (log-rank p=0·10). The corresponding median EFS was 1·0 (0·7–1·4), 2·2 (1·1–6·0), 1·0 (0·6–1·5), and 1·0 (0·6–2·0) years (log-rank p=0·005). Forest plots for EFS, overall survival, time to recurrence, and death from head and neck cancer for various treatment comparisons are shown in the webappendix. Compared with radiotherapy alone, SIM alone was associated with an improvement in EFS (HR 0·72, 99% CI 0·53–0·96; p=0·004), although the effect on overall survival was not significant (0·82, 0·60–1·11; p=0·09). Estimated 5-year overall survival and EFS in the SIM-alone group were 50% (95% CI 39–59) and 42% (32–52; the corresponding rates in the radiotherapy alone group were 39% [31–48] and 24% [17–31]). There was no evidence of a survival benefit for either SUB alone (overall survival HR 1·10 [0·81–1·48]; p=0·42) or SIM+SUB (overall survival HR 1·06 [0·78–1·44]; p=0·62). Comparing four with two courses of chemotherapy (ie, SIM+SUB *vs* either SIM alone or SUB alone) did not show a survival advantage (webappendix). Adding SUB to SIM seemed to reduce the benefit of SIM, producing a higher rate of death and events: compared with SIM alone, overall survival (HR 1·29; 99% CI 0·92–1·81**;** p=0·049) and EFS (HR 1·27, 0·92–1·75**;** p=0·06). HR from an analysis of the main effects associated with a 2×2 factorial trial (ie, any SIM *vs* no SIM, and any SUB *vs* no SUB [test for the interaction between SIM and SUB p=0·12], as well as SIM alone *vs* SUB alone, and radiotherapy alone *vs* SIM alone or SUB alone), are shown in the webappendix.

Adding chemotherapy to radiotherapy was not effective in patients who had undergone surgery previously. The median overall survival was 4·6 (99% CI 2·2–7·6) and 5·0 (1·8–8·0) years in those allocated to SIM alone or radiotherapy alone, respectively (HR 0·94, 99% CI 0·64–1·40; p=0·70). The median EFS was 3·0 (99% CI 1·2–5·6) years and 3·7 (1·1–5·9) years in the SIM-alone and radiotherapy-alone group (HR 1·03, 99% CI 0·71–1·49; p=0·85). The median overall survival associated with SIM alone was similar to the corresponding SIM group without previous surgery (4·6 *vs* 4·7 years), but the median EFS was higher (3·0 *vs* 2·2 years).

Estimated absolute risk differences for overall survival, EFS, and recurrence at 5 and 10 years after randomisation are shown in table 4. Compared with radiotherapy alone, for every 100 patients given SIM alone, there could be 10·6 fewer patients who have a recurrence, new tumour, or die by 10 years after treatment (99% CI 1·1–21·2 fewer); equivalent to a number needed to treat of nine patients to avoid one event at 10 years. Similar effects were seen for overall survival. There could be 7·1 fewer deaths at 10 years (99% CI 18·4 fewer to 3·5 more) among 100 patients treated with SIM alone (number needed to treat of 14). No benefit was seen with SUB alone or SIM+SUB, consistent with the other results.

**Table 4:** Estimated absolute risk differences (99% CI) 5 and 10 years after randomisation, among patients without previous surgery

	**Deaths (any cause)**		**Events (recurrence, new tumour, or death)**		**Recurrences**	
	5 years	10 years	5 years	10 years	5 years	10 years
SIM alone *vs* radiotherapy alone	−7·3 (−17·8 to 3·9)	−7·1 (−18·4 to 3·5)	−12·1 (−22·9 to −1·4)	−10·6 (−21·2 to −1·1)	−11·0 (−22·8 to 1·7)	−11·1 (−23·0 to 1·7)
Any SIM *vs* radiotherapy alone	−2·6 (−11·7 to 6·8)	−2·5 (−11·7 to 5·9)	−7·4 (−16·4 to 1·2)	−6·3 (−14·7 to 1·0)	−6·7 (−16·7 to 3·8)	−6·7 (−16·7 to 3·8)
SUB alone *vs* radiotherapy alone	3·4 (−7·5 to 14·3)	3·1 (−7·3 to 11·8)	1·3 (−8·8 to 10·1)	1·1 (−7·5 to 7·5)	2·0 (−10·0 to 13·6)	2·0 (−10·0 to 13·4)
Any SUB *vs* radiotherapy alone	2·8 (−6·4 to 12·1)	2·6 (−6·2 to 10·2)	−0·7 (−9·3 to 7·2)	−0·5 (−8·0 to 5·5)	0·1 (−10·0 to 10·2)	0·1 (−10·1 to 10·1)
Any SIM *vs* no SIM	−8·4 (−12·2 to 4·0)	−8·1 (−14·1 to −2·3)	−8·1 (−15·9 to −0·5)	−7·0 (−14·4 to −0·4)	−7·4 (−16·3 to 1·8)	−7·4 (−16·4 to 1·8)
Any SUB *vs* no SUB	5·9 (−2·2 to 14·0)	5·4 (−2·2 to 12·2)	4·3 (−3·4 to 11·5)	3·6 (−3·0 to 9·1)	4·7 (−4·3 to 13·7)	4·7 (−4·3 to 13·6)
SIM+SUB *vs* SIM alone	9·4 (2·9 to 21·2)	8·4 (−2·8 to 17·1)	8·7 (−3·1 to 20·1)	7·8 (−3·0 to 16·4)	9·0 (−4·6 to 22·8)	9·0 (−4·7 to 22·8)
SIM+SUB *vs* SUB alone	−1·3 (−13·3 to 10·3)	−1·3 (−13·1 to 9·2)	−4·4 (−15·8 to 5·8)	−3·8 (−14·4 to 4·6)	−3·9 (−17·0 to 9·1)	−3·9 (−17·1 to 8·9)

SIM=radiotherapy with two courses of chemotherapy given simultaneously on days 1 and 14 of radiotherapy. SUB= radiotherapy with two courses of chemotherapy given 14 and 28 days after completing radiotherapy. Negative risk differences indicate that the event rate is lower than in the comparison group (ie, more beneficial); positive differences indicate that the event rate is higher. Any SIM=SIM or SIM+SUB. Any SUB=SUB or SIM+SUB. The differences were estimated by applying the hazard ratios (HR) and CI to the 5-year or 10-year rate in the comparison group. Difference=exp[HR×ln(R1)]−R1. For example, HR could be the HR when comparing SIM to RT alone, and R1 the cumulative survival rate for the “RT alone” group at 10 years.

The percentages of patients who had a significant toxicity during treatment requiring hospitalisation in patients without previous surgery was 11% (25/233) in patients allocated to radiotherapy alone, 28% (47/166) in patients allocated to SIM alone, 12% (19/160) in patients allocated to SUB alone, and 36% (55/154) in patients allocated to SIM+SUB (table 5). The toxicity rates did not differ substantially between the type of chemotherapy used (methotrexate or VBMF; table 5). Although 28% of patients in the SIM-alone group had an acute toxicity, compliance to chemotherapy was high (92%, 152/166; table 2), probably because being in hospital would have provided them with the appropriate care to continue treatment. For patients who had undergone previous surgery, SIM alone was associated with a doubling in the acute toxicity rate: 20% (24/118) SIM alone versus 9% (12/135) radiotherapy alone. Patients in the SIM+SUB group who experienced acute toxicity during chemoradiation were less likely to complete their allocated chemotherapy regimen (table 2).

**Table 5:** Toxicities reported during treatment and at least 6 months after randomisation according to trial group

		**No surgery**				**Surgery**	
		Radiotherapy alone (N=233)	SIM alone (N=166)	SUB alone (N=160)	SIM+SUB (N=154)	Radiotherapy alone (N=135)	SIM alone (N=118)
**During treatment (requiring hospitalisation)**							
Mucositis		23 (10)	43 (26)	14 (9)	46 (30)	9 (7)	20 (17)
Candidiasis		8 (3)	8 (5)	2 (1)	7 (5)	1 (<1)	2 (2)
Renal failure		0	0	0	2 (1)	0	0
Septicaemia		0	1 (<1)	0	2 (1)	0	1 (1)
Reaction to methotrexate/VBMF		0	0	2 (1)	2 (1)	0	2 (2)
Radiation pharyngitis		0	0	0	0	2 (1)	0
Transient ischaemic attack		0	0	1 (<1)	0	1 (<1)	0
Other		2 (<1)	3 (2)	2 (1)	11 (7)	1 (<1)	3 (3)
According to chemotherapy							
	VBMF*	n/a	16 (35)	4 (9)	20 (45)	n/a	5 (18)
	Methotrexate*	n/a	31 (26)	15 (13)	35 (32)	n/a	19 (21)
Any toxicity†		25 (11)	47 (28)	19 (12)	55 (36)	12 (9)	24 (20)
**Occurring at least 6 months after randomisation**							
Xerostomia		6 (3)	2 (1)	2 (1)	3 (2)	2 (1)	3 (3)
Trismus		0	0	0	0	0	2 (2)
Mucositis		1 (<1)	0	1 (<1)	0	1 (<1)	0
Dysphagia		0	3 (2)	1 (<1)	2 (1)	1 (<1)	5 (4)
Fibrosis		0	1 (<1)	0	0	0	2 (2)
Stricture		1 (<1)	0	0	3 (2)	4 (3)	3 (3)
Other		5 (2)	4 (2)	5 (3)	2 (1)	2 (1)	4 (3)
According to chemotherapy							
	VBMF*	n/a	6 (13)	2 (4)	5 (11)	n/a	3 (11)
	Methotrexate*	n/a	4 (3)	5 (4)	4 (4)	n/a	10 (11)
Any toxicity†		13 (6)	10 (6)	7 (4)	9 (6)	10 (7)	13 (11)

SIM=radiotherapy with two courses of chemotherapy given simultaneously on days 1 and 14 of radiotherapy. SUB= radiotherapy with two courses of chemotherapy given 14 and 28 days after completing radiotherapy. n/a=not applicable. Data are n(%). VBMF=vincristine, bleomycin, methotrexate, and fluorouracil. Patients could have more than one toxicity.

* Expressed as a percentage of those allocated to VBMF or methotrexate (webappendix).

† Each patient counted once.

Significant late toxicity occurring 6 months or more after randomisation was reported in 62 patients in total; 44% (n=27) of first notifications occurring in months 6–11·9, and 24% (n=15) occurring in months 12–24. The rates were similar between the trial groups: 6% (13/233) among patients without surgery allocated to radiotherapy alone, 6% (10/166) for those allocated to SIM alone, 4% (7/160) for those allocated to SUB alone, and 6% (9/154) for those assigned to SIM+SUB (table 5); and 7% (10/135) and 11% (13/118) among patients who had undergone surgery allocated to radiotherapy alone or SIM alone, respectively.

There were 12 treatment-related deaths, largely associated with dehydration, necrosis, mucositis, dysphagia, and renal failure. Deaths were broken down by treatment group as follows: SIM alone (n=1), SUB alone (n=2), SIM+SUB (n=8), and radiotherapy alone (n=1). The first six deaths were sent for independent review, after which clearer instructions were given to patients regarding adequate hydration and, in some centres, the avoidance of non-steroidal anti-inflammatory agents (recognised to reduce glomerular filtration rate). Among the eight deaths in the SIM+SUB group, two occurred during SIM treatment, five occurred after completing radiotherapy but before starting SUB (four of which occurred at least 1 month after finishing SIM), and one occurred during SUB.

## Discussion

The UKHAN1 trial is one of the largest in head and neck cancer, and, to our knowledge, the only study to examine the timing of chemotherapy (concurrent or maintenance) in a factorial design. Compared with radiotherapy alone, two courses of non-platinum chemotherapy given concurrently with radiotherapy (SIM alone) in patients without previous surgery significantly extended EFS by 1·2 years (median EFS 2·2 *vs* 1·0 years; p=0·004). The difference in overall survival of 2·1 years (median 4·7 *vs* 2·6 years, p=0·09) was not statistically significant, probably because there were fewer overall survival events compared with EFS in the analysis of SIM alone versus radiotherapy alone (293 deaths *vs* 322 EFS events) and the effect size was smaller (HR of 0·82 *vs* 0·72). The outcomes for SIM alone were achieved with only one treatment-related death (0·6%), high compliance (92%), and acceptable toxicity rate (28%) during treatment. Importantly, we are able to report the long-term benefits of SIM. Among every 100 patients treated with SIM alone, there were an estimated 10·6 fewer patients with a recurrence, new tumour, or death, 10 years later, compared with radiotherapy alone. Concurrent chemoradiotherapy used in our study did not benefit patients who had undergone previous surgery, and the acute toxicity rate doubled.

Chemotherapy after radiotherapy was ineffective, which could be due in part to the higher proportion of patients who did not comply with treatment. Reasons for non-compliance (table 2) were largely because of toxicity, deaths, or withdrawals (perhaps because some felt too unwell after radiotherapy to continue with therapy). Chemotherapy non-compliance was highest in the SIM+SUB group, and might also be due to patients not wanting to continue treatment after two courses of chemotherapy, possibly because they had completely responded after simultaneous chemoradiation, or were too unwell to continue. Furthermore, overall survival and EFS were worse in the SIM+SUB group than with SIM alone.

UKHAN1 centres were allowed to choose the radiotherapy regimen. This is because when the trial was designed in 1989 there was concern over the potential toxic interaction between radiotherapy and one or more of the chemotherapy groups. Data were emerging that gaps in radiotherapy were detrimental in patients with squamous-cell carcinomas, and so should be avoided. We therefore allowed a range of regimens that would be tolerated by most patients, making them less likely to require rests from radiotherapy due to chemotherapy toxicity. Furthermore, this was a pragmatic trial, so we wanted centres to give their normal radiotherapy to encourage clinicians to participate, and therefore the doses used represented those given in routine practice at the time. All the radiotherapy regimens were radical doses, and there was no evidence that the radiotherapy regimen used affected EFS (webappendix).

There is variation in how patients with head and neck cancer are treated, with no established and agreed policy on chemoradiation, including for patients judged unfit for platinum therapy. After the first MACH-NC meta-analysis of chemotherapy in head and neck cancer,^6^ the benefit from routine chemotherapy remained unclear. However, in the recent update, based on 9615 patients and including older data from UKHAN1, concomitant chemotherapy led to statistically significant reductions in deaths and recurrences,^7^ with point estimates similar to those from UKHAN1: HR for overall survival 0·81 in MACH-NC versus 0·82 (99% CI 0·60–1·11) in UKHAN1, and HR for EFS 0·79 in MACH-NC versus 0·72 (99% CI 0·53–0·96**)** in UKHAN1 (MACH-NC used recurrence-free survival instead of EFS). MACH-NC also confirmed that adjuvant chemotherapy is ineffective (overall survival HR 0·99, 95% CI 0·89–1·10, in 2567 patients). The review was based on adding chemotherapy to any loco-regional treatment (ie, a mixture of surgery, no surgery, radiotherapy using standard or hyperfractionated regimens, or pre-operative radiotherapy). UKHAN1 clearly separates patients according to whether they had surgery or not, showing that concurrent non-platinum chemotherapy used in the trial was more effective than radiotherapy alone in the non-surgical group, but not in the group that had undergone previous surgery. Additionally, our results are based on patients with a median follow-up of 10 years, compared with 5·6 years in the meta-analysis. Finally, our study provides direct randomised evidence on the timing of chemotherapy (ie, comparing concurrent with maintenance chemotherapy).

The MACH-NC meta-analysis showed that concurrent chemoradiation should now be the routine treatment of choice for all patients with non-surgically treated advanced head and neck cancer. The UKHAN1 study confirms this, but it also shows that a long-term benefit in terms of recurrence and deaths could be achieved with non-platinum agents that are inexpensive, relatively easy to deliver, and have lower toxicity than platinum therapies. Very few randomised head and neck cancer trials have reported such long-term effects of chemo-radiotherapy, and we are not aware of any that have also investigated concurrent platinum treatment.

Cisplatin used concurrently with radical radiotherapy has been shown to be better than radiotherapy alone: in an EORTC trial (334 post-surgical patients with locally advanced disease), the risk of death was reduced by 30% (p=0·02).^12^ EFS was improved with cisplatin in a RTOG trial (459 high-risk patients who had complete resection), with a decrease in risk (HR 0·78, 95% CI 0·61–0·99**;** p=0·04).^13^ However, acute toxicity rates were much higher in those receiving chemotherapy (77% RTOG and 41% EORTC), compared with that in our own trial using VBMF or methotrexate (28%). Although cisplatin-based regimens are often used in the US, the MACH-NC meta-analysis did not find a survival difference according to type of chemotherapy (p=0·42). In trials of poly-chemotherapy, the HR for death was 0·75 (95% CI 0·67–0·84; using both fluorouracil and platinum), 0·83 (0·74–0·94; using either fluorouracil or platinum), and 0·73 (0·52–1·01; using chemotherapy other than fluorouracil or platinum); the UKHAN1 estimate was 0·82.

Newer agents such as taxanes and targeted therapies might improve on the longer-established doublet of cisplatin and fluorouracil.^14^, ^15^, ^16^ Bonner and colleagues were the first to show the value of cetuximab in advanced head and neck cancer, with a 30% reduction in the number of patients who progress or die (in a trial of 424 patients).^16^ Similar therapy combinations continue to be investigated. However, the potential late morbidity of combination chemoradiotherapy, including secondary tumours, should be monitored. Our 10-year follow-up is reassuring for the regimens used in the UKHAN1 study, and should encourage other trials to follow patients for many years.

Hyperfractionated radiotherapy, involving an increase in the daily number of administered doses, is currently difficult for many UK oncology departments to deliver, so the simpler two courses of chemotherapy with only one fraction per day using standard regimens seems more feasible. As with improved outcomes at other sites such as the cervix, anus and lung, radical simultaneous chemoradiation therapy for SCC of the head and neck is an effective way of treating these life-threatening and disabling diseases.

Human papillomavirus (HPV) status was not obtained during the trial because it was not recognised as being a useful factor in head and neck cancer at the time the study was designed. However, data are beginning to emerge suggesting that patients with HPV-positive tumours have a better prognosis.^17^ Although most evidence comes from observational studies, the effects have been seen in retrospective analyses of randomised trials.^18^, ^19^ We plan to examine HPV status and its possible effect on outcome in the UKHAN1 trial in the future.

In summary, UKHAN1 showed that patients with head and neck cancer who had undergone previous surgery did not benefit from the addition of chemotherapy to adjunctive post-operative radiotherapy. However, there was a clear benefit on recurrences and deaths associated with two courses of simultaneous non-platinum chemoradiotherapy in patients who had not undergone previous surgery, and this benefit persisted after a long follow up. Non-platinum-based chemotherapy could be considered an alternative to platinum-based regimens, as well as an effective therapy for patients who are judged to be unfit for cisplatin. With this particular group of patients, characteristically with multiple comorbidity and relatively low compliance to chemotherapy, the inability to tolerate platinum-based regimens is often a serious barrier to radical chemoradiation. Moreover, the avoidance of local recurrence, with its potentially devastating consequences for the patient's quality of life, is of particular importance in this group, many of whom suffer from social deprivation and poor domestic support. Improving EFS reduces the number of patients who later need radical salvage surgery, which can be associated with long-term or permanent disfigurement, impaired function (eg, ability to speak or eat easily), or social exclusion.^20^ Because this is a high risk and generally unfit patient group, many of whom are excessive users of alcohol and tobacco throughout treatment, the availability of a relatively simple, inexpensive and low toxicity chemoradiation regimen considerably improves the likelihood of completing treatment, essential for improving the chance of cure.

## Web Extra Material

Supplementary webappendix
