# Supplementary material for: Chemoradiotherapy for locally advanced head and neck cancer: 10-year follow-up of the UK Head and Neck (UKHAN1) trial
Source: Lancet Oncol. 2009 Sep 9;10(9):872–6. doi: 10.1016/S1470-2045(09)70306-7 (PMC2806549; doi:10.1016/S1470-2045(09)70306-7)
Supplement: Supplementary webappendix [file mmc1.pdf]

THE LANCET Oncology

Supplementary webappendix

This webappendix formed part of the original submission and has been peer reviewed. We post it as supplied by the authors.

Supplement to: Tobias JS, Monson K, Gupta N, et al, on behalf of the UK Head and Neck Cancer Trialists' Group. Chemoradiotherapy for locally advanced head and neck cancer: 10-year follow-up of the UK Head and Neck (UKHAN1) trial. *Lancet Oncol* 2009; published online October 28. DOI:S1470-2045(09)70306-7.

Treatment comparison	HR (99% CI)	No. of events/ subjects
**No Surgery**		
Any chemo vs RT alone	0.88 (0.70, 1.10)	595/713
SIM alone vs RT alone	0.72 (0.53, 0.96)	322/399
Any SIM# vs RT alone	0.81 (0.63, 1.04)	454/553
SUB alone vs RT alone	1.04 (0.78, 1.38)	338/393
Any SUB* vs RT alone	0.98 (0.77, 1.25)	470/547
(SIM+SUB) vs RT alone	0.92 (0.69, 1.23)	329/387
(SIM alone+SUB alone) vs RT	0.85 (0.67, 1.09)	463/559
SIM alone vs SUB alone	0.68 (0.50, 0.94)	266/326
SIM vs No SIM	0.80 (0.64, 0.99)	595/713
SUB vs No SUB	1.13 (0.91, 1.39)	595/713
(SIM+SUB) vs SIM alone	1.27 (0.92, 1.75)	257/320
(SIM+SUB) vs SUB alone	0.88 (0.64, 1.20)	273/314
**Surgery**		
SIM alone vs RT alone	1.03 (0.71, 1.49)	192/253

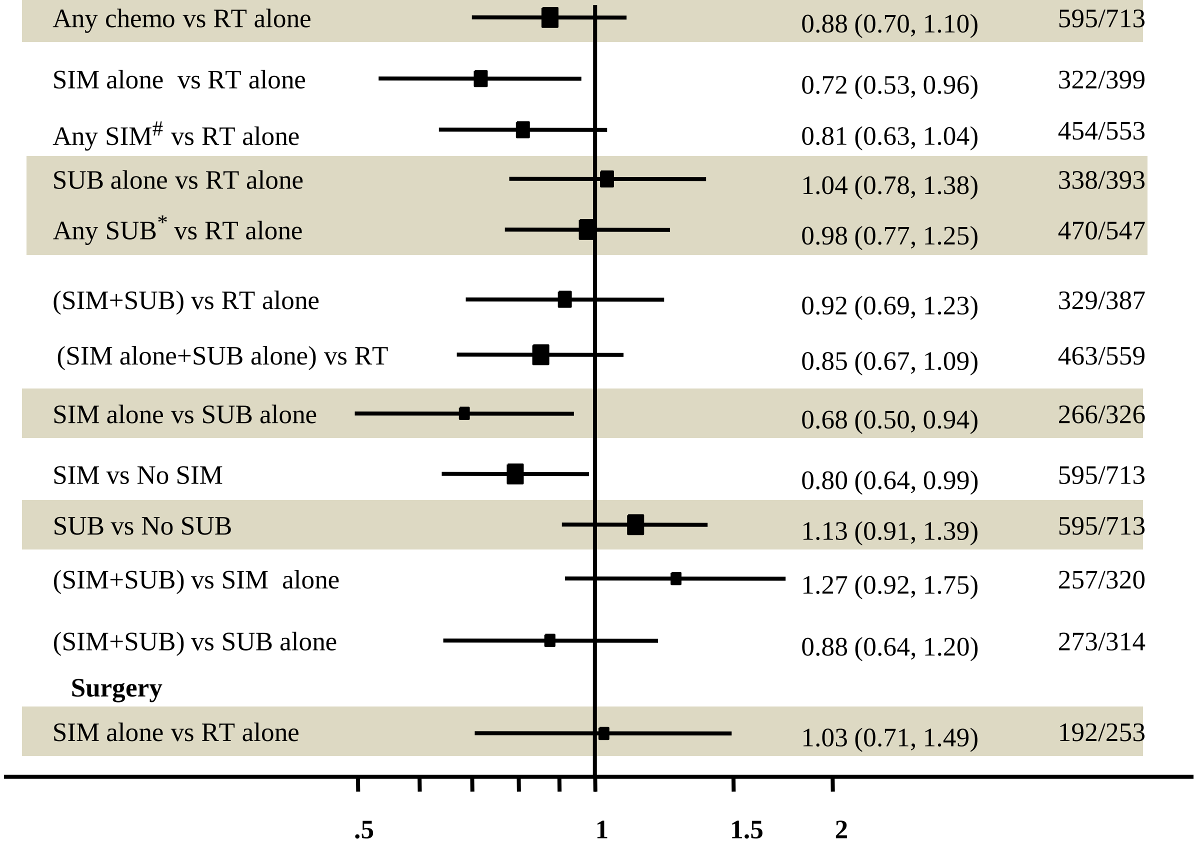

SIM & SIM+SUB
* SUB & SIM+SUB

Appendix Figure 1. Hazard ratios (and 99% confidence intervals) for EFS for specified treatment comparisons

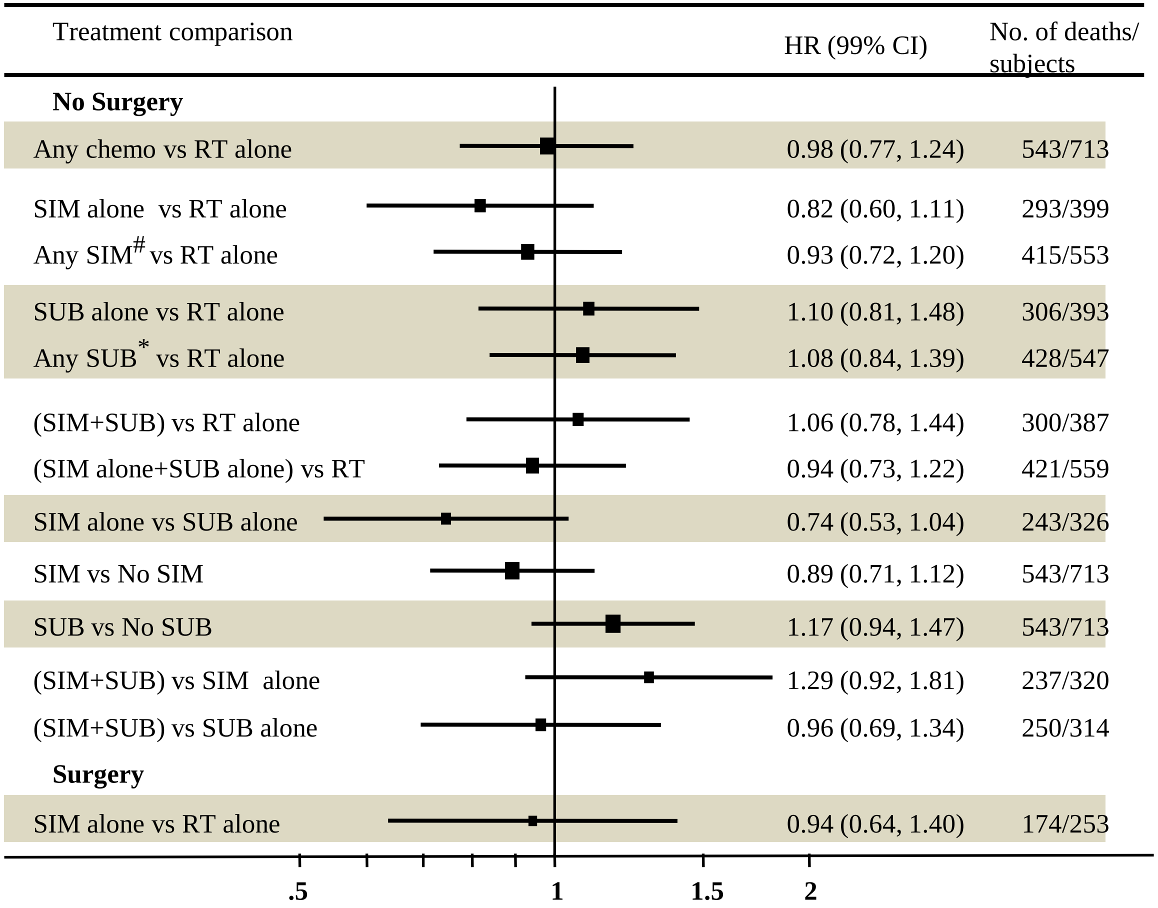

SIM & SIM+SUB
* SUB & SIM+SUB

Appendix Figure 2. Hazard ratios for overall survival according to specified treatment comparisons

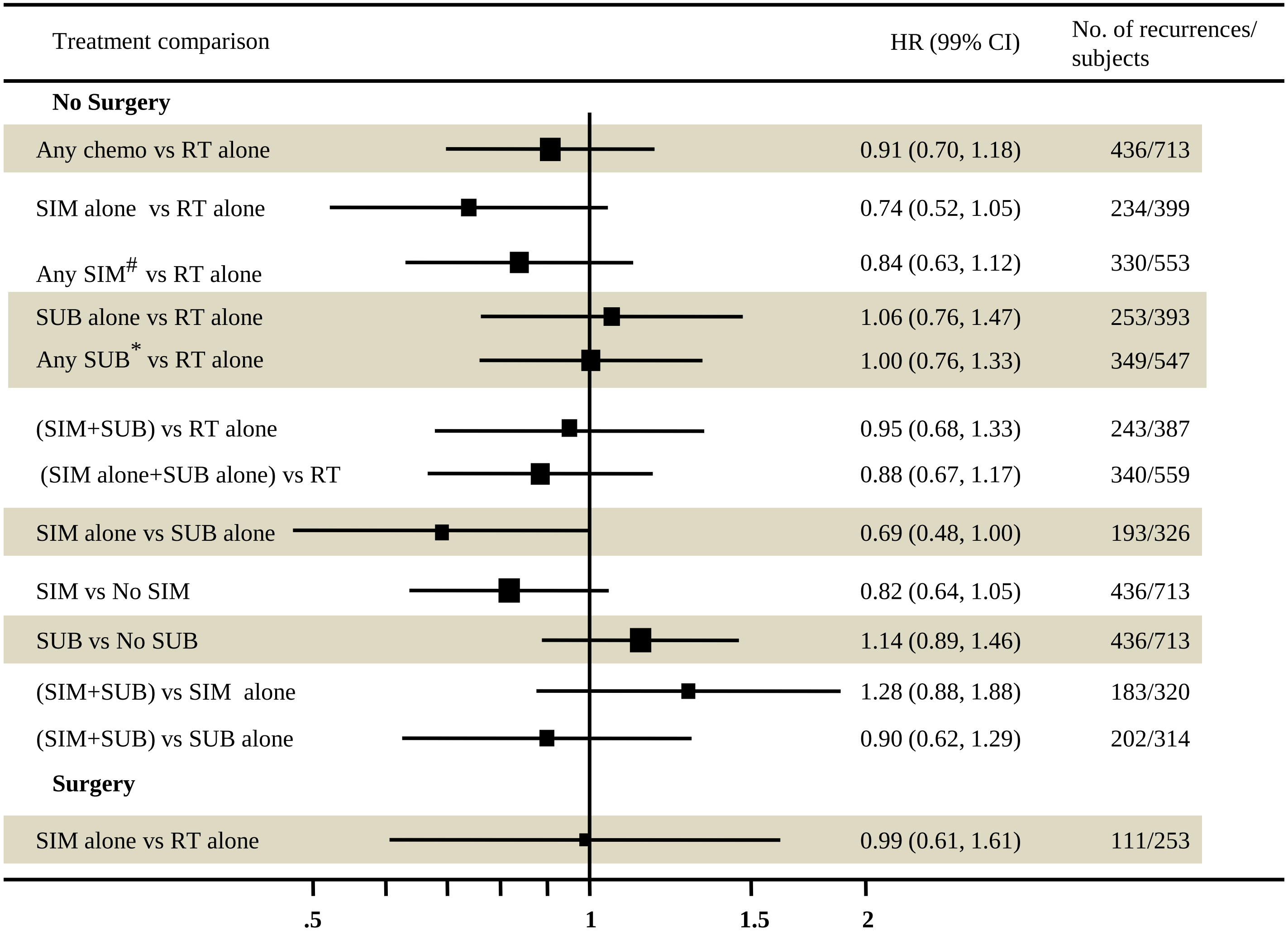

SIM & SIM+SUB
* SUB & SIM+SUB

Appendix Figure 3. Hazard ratios (and 99% confidence intervals) for time to recurrence for specified treatment comparisons

Treatment comparison	HR (99% CI)	No. of deaths/ subjects
**No Surgery**		
Any chemo vs RT alone	1.00 (0.75, 1.34)	370/713
SIM alone vs RT alone	0.88 (0.61, 1.27)	203/399
Any SIM# vs RT alone	0. 91(0.66, 1.24)	275/553
SUB alone vs RT alone	1. 22 (0.85, 1.73)	215/393
Any SUB* vs RT alone	1.08 (0.79, 1.47)	287/547
(SIM+SUB) vs RT alone	0.94 (0.64, 1.38)	192/387
(SIM alone+SUB alone) vs RT	1.03 (0.76, 1.40)	298/559
SIM alone vs SUB alone	0.72 (0.49, 1.06)	178/326
SIM vs No SIM	0.84 (0.64, 1.10)	370/713
SUB vs No SUB	1.14 (0.87, 1.49)	370/713
(SIM+SUB) vs SIM alone	1. 06 (0.70, 1.62)	155/320
(SIM+SUB) vs SUB alone	0.77 (0.51, 1.15)	167/314
**Surgery**		
SIM alone vs RT alone	0.93 (0.58, 1.49)	121/253

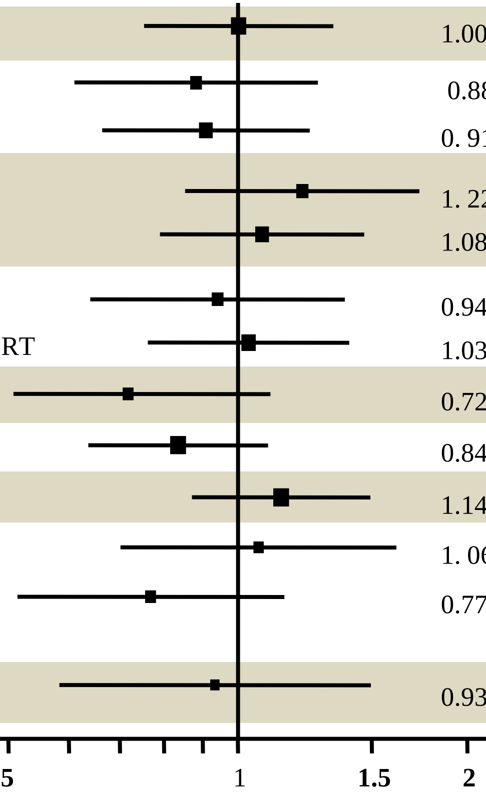

#SIM & SIM+SUB
*SUB & SIM+SUB

Appendix Figure 4. Hazard ratios (and 99% confidence intervals) for death from head and neck cancer for specified comparisons

Appendix Table 1: Chemotherapy and radiotherapy regimens according to trial arm

Centres giving	No surgery (N=713)				Surgery (N=253)	
	RT alone (N=233)	SIM alone (N=166)	SUB alone (N=160)	SIM+SUB (N=154)	RT alone (N=135)	SIM alone (N=118)
Chemotherapy*				No. patients (%)		
Methotrexate (24 centres)	172 (74)	120 (72)	113 (71)	110 (71)	101 (75)	90 (76)
VBMF (12 centres)	61 (26)	46 (28)	47 (29)	44 (29)	34 (25)	28 (24)
Radiotherapy						
SECOG	110 (47)	80 (48)	76 (48)	71 (46)	52 (39)	42 (36)
Manchester	49 (21)	33 (20)	35 (22)	33 (21)	1 (<1)	2 (2)
'Birmingham/Edinburgh' (and a few others)	71 (30)	51 (31)	44 (28)	46 (30)	81 (60)	73 (62)
Other regimens	3 (1)	2 (1)	5 (3)	4 (3)	1 (<1)	1 (<1)

* not all patients who were due to receive chemotherapy did so

There were a total of 34 centres. Two centres gave both VBMF and Methotrexate depending on the consultants
VBMF: vincristine, bleomycin, methotrexate and fluorouracil

The total numbers of patients randomised to receive methotrexate or VBMF were 433 or 165 respectively, but of these 417 and 153 actually received these treatments (most of the others either died or declined to have chemotherapy).

Among patients without prior surgery, the hazard ratios for event-free survival (EFS) for SIM, SUB and SIM+SUB (each compared to RT alone) were 0.72 (95% CI 0.56 to 0.93), 1.03 (95% CI 0.81 to 1.31), and 0.92 (95% CI: 0.72 to 1.18) for those receiving methotrexate, and 0.75 (95% CI 0.53 to 1.06), 1.19 (95% CI 0.87 to 1.62), and 0.98 (95% CI 0.69 to 1.39) for those receiving VBMF. Among patients with prior surgery, the hazard ratios for SIM vs RT alone were 0.87 (95% CI 0.63 to 1.21) and 0.86 (95% CI: 0.55 to 1.33) for those receiving methotrexate and VBMF respectively.

The hazard ratios for EFS for SIM alone vs RT alone (patients without prior surgery) were 0.77 (95% CI 0.56-1.07) for SECOG; 0.83 (95% CI 0.51-1.33) for Manchester regimen; and 0.55 (95% CI 0.35-0.87) for Birmingham/Edinburgh regimen. All confidence intervals include the overall hazard ratio of 0.72, indicating no evidence of a differential treatment effect according to RT regimen used.

Appendix Table 2. Disease status at 6 months post-randomisation

Disease status	**No surgery (N=713)**				**Surgery (N=253)**		**Total (N=966)**
	RT alone (N=233)	SIM alone (N=166)	SUB alone (N=160)	SIM+SUB (N=154)	RT alone (N=135)	SIM alone (N=118)	
	No. patients (%)						
Died	17 (7)	8 (5)	18 (11)	28 (18)	16 (12)	8 (7)	95 (10)
Never disease-free	20 (9)	5 (3)	9 (6)	11 (7)	4 (3)	0 (0)	49 (5)
Recurrence	35 (15)	24 (14)	19 (12)	12 (8)	11 (8)	16 (14)	117 (12)
New tumour	1 (<0.5)	0	0	1 (1)	1 (1)	0	3 (<0.5)
Salvage treatment	13 (6)	5 (3)	6 (4)	5 (3)	0	1 (1)	30 (3)
Disease-free at 6 month	146 (63)	122 (73)	106 (66)	95 (62)	102 (76)	91 (77)	662 (69)
Data missing	1 (<0.5)	2 (1)	2 (1)	2 (1)	1 (1)	2 (2)	10 (1)
**Total**	**233**	**166**	**160**	**154**	**135**	**118**	**966**

All patients were prescribed radiotherapy
